# The Longitudinal and Reciprocal Associations Among Maternal Aggravation, Verbal Aggression, and Internalizing Problems from Childhood to Adolescence

**DOI:** 10.3390/bs16020201

**Published:** 2026-01-30

**Authors:** Kayla Stange-Bacher, Ming Cui

**Affiliations:** Department of Human Development and Family Science, Florida State University, Tallahassee, FL 32306, USA; kstange@fsu.edu

**Keywords:** internalizing problems, maternal aggravation, verbal aggression, childhood, adolescence, FFCWS

## Abstract

Parents have a major influence on a child’s wellbeing, including the development of internalizing problems in their children. Furthermore, the influences between parents and their children could be reciprocal. This study examined the longitudinal, reciprocal association between maternal aggravation and child internalizing problems from childhood to adolescence and the potential mediating role of maternal verbal aggression. Using a sample of approximately 5000 mothers across four waves from the Future of Families & Child Wellbeing Study (FFCWS), results from a cross-lagged autoregressive model suggested that maternal aggravation was related to child internalizing problems from childhood to adolescence, whereas child internalizing problems were related to maternal aggravation only during early childhood. No mediation effect through maternal verbal aggression was found. Taken together, these findings highlight the long-term and direct impact of maternal aggravation on child internalizing problems, which has important implications for family researchers and practitioners.

## 1. Introduction

With the rise in internalizing problems, poor mental health among children and adolescents is a substantial public health concern. Internalizing problems in childhood primarily involves anxious and depressive symptoms (Bayer et al., 2006). According to The National Survey of Children’s Health (2024), anxiety and depression are the most common mental health issues among children and adolescents (also see Centers for Disease Control and Prevention [CDC], 2023; Chiu et al., 2016). The Centers for Disease Control and Prevention (CDC) estimated that 7.1% of children and adolescents ages 3–17 have been diagnosed with an anxiety disorder and 3.2% with depressive disorder (Centers for Disease Control and Prevention [CDC], 2023).

Given the rise in internalizing problems among children and adolescents, parents play an increasingly important role in affecting how their children develop into adolescence and beyond (Gawlik & Melnyk, 2022). Being a parent, although rewarding at times, can be difficult, especially as a low-income, single mother. Roughly one-in-four (41%) of parents have reported that being a parent is tiring, and 29% reported that being a parent is stressful (Minkin & Horowitz, 2023). When parents are stressed, the ways they respond emotionally towards their child can be negatively impacted (Nelson et al., 2009). When parents are under stress, and their children are around, some may display healthy emotional regulation skills, which have been shown to contribute to fewer internalizing problems in children (Zimmer-Gembeck et al., 2021). On the other hand, some may display maladaptive emotional regulation skills, which could contribute to hostile family interactions that promote more internalizing problems in children (Zimmer-Gembeck et al., 2021; Zitzmann et al., 2023). Parents with difficulties regulating their emotions often engage in negative parenting behaviors (Zimmer-Gembeck et al., 2021), which can include showing aggravation and verbal aggression towards their children.

Parental aggravation is defined as feelings of frustration and annoyance with tainted perceptions of their children stemming from the high demands of being a parent (Bronte-Tinkew et al., 2009). From a 1997 sample of American family households across the nation conducted by the National Survey of America’s Families (NSAF), 9% of children lived with a parent who had reported feeling highly aggravated (Moore & Ehrle, 1999). Harshness and anger are also common traits of parental aggravation, which can manifest into strained parent–child relationships and poorer developmental trajectories in children, including an increase in internalizing problems (Jennings & Deitz, 2007; Park et al., 2023). Verbal aggression is defined as repetitive, harmful verbalized behaviors that are commonly unprovoked (Reichman et al., 2001). Verbal aggression, which could be due to parental aggravation, can take the forms of insulting, swearing, and/or threatening (Achenbach, 1992). From surveying 3346 American parents, 63% reported one or more instances of verbal aggression towards their children (Vissing et al., 1991). In addition, it was found that children who endured parental verbal aggression exhibited higher rates of physical aggression, delinquency, and interpersonal issues (Vissing et al., 1991). With this insight, the role of parental aggravation and subsequent verbal aggression in child wellbeing needs to be further addressed and studied.

Previous developmental studies have found that individuals who have high levels of internalizing problems during childhood are likely to experience a continuation of these problems going into adolescence (Cohen et al., 2017). Consequences of these trajectories include interpersonal issues, substance abuse, poorer physical health, and worsened financial stability (Copeland et al., 2014; Morales-Muñoz et al., 2023). With these negative continuous trends, the question arises about who and what influences children to experience these internalizing problems at different severity levels, with a specific interest surrounding anxiety and depression symptoms. Research emphasis is put on family factors, with maternal aggravation promoting verbal aggression, therefore putting them at a higher risk for developing internalizing problems.

Despite empirical studies pertaining to negative parenting and its effects on children, there is limited research surrounding how maternal aggravation could transform into verbal aggression towards their children, which could then lead to an increase in childhood internalizing problems. Equally important, there are no known studies pertaining to the reciprocal processes of how children who exhibit internalizing problems could influence their parents to exhibit verbal aggression, which could then transform into aggravation towards them. Taken together, this study aims to study the longitudinal associations among maternal aggravation, verbal aggression, and internalizing problems from childhood to adolescence and to further explore the possible reciprocal effects between these maternal behaviors and child wellbeing.

### 1.1. Theoretical Perspectives

Poor parental attitudes and behaviors can be detrimental to child outcomes regarding psychological and social wellbeing (Moore & Ehrle, 1999; Murphey et al., 2014). Family socialization theory (Murphey et al., 2014) applies to this phenomenon. In broad terms, socialization refers to how individuals are taught characteristics needed to function in society through interacting with and observing others; these characteristics can involve behaviors, values, standards, and motives (Bugental & Grusec, 2007). From a human development standpoint, socialization begins with parent–child relationships. Families (deemed as primary agents) have the first and strongest contact with the child (Grusec, 2011; Nickerson, 2024). As children often adopt characteristics from their strongest contact, these first contacts are critical to socioemotional development. Parents have direct influences over their children’s self-regulation, emotion, thinking, and behavior (Grusec, 2002). According to Bandura (1973), most human thought processes and behaviors are learned through those surrounding them; therefore, through observing and interacting with parents, children could develop either positive or negative thought and behavior processes depending on the socialization provided by their parents. Since family socialization theory is loosely based on social learning theory (Bandura, 1973), we can attest that children learn and model behavior shown by their parents. For example, children who observe their parents responding maladaptively to stress learn that showing excessive aggravation is acceptable, resulting in them adopting those poor emotional regulation techniques when feelings of aggravation arise (Kalmuss & Seltzer, 1989). This is critical, as children with poorer emotional regulation skills are at higher risk for developing internalizing problems (Loevaas et al., 2018).

Family systems theory (FST) (Bowen, 1966) emphasizes that families are interdependent units where all their members are connected emotionally (The Bowen Center for the Study of the Family [BCSF], 2023b). All members within the family are often affected by other members’ thoughts, feelings, and/or actions. When one family member displays feelings such as distress, other family members within that unit may feel increasingly distressed as well (Bowen, 1966). Parents tend to be one of the most emotionally influential members in the family unit, especially in relation to children’s mental wellbeing (National Academies of Sciences, Engineering, and Medicine, 2016). Based on family projection processes (FPP) (The Bowen Center for the Study of the Family [BCSF], 2023a), which is a component of FST, parents who provide warm, non-distressing environments for their children help minimize the risks of their children developing mental health issues. In comparison, parents who provide hostile, distressing environments for their children, perhaps through showing aggravation and verbal aggression, increase the risks of their children developing internalizing problems such as anxiety and depression (Bayer et al., 2006). On the other hand, problems in children could also lead to negative reactions from their parents, including aggravation and aggression. This could be due to spillover effects between the parents and their children regarding poor parental functioning and poor child functioning (The Bowen Center for the Study of the Family [BCSF], 2023a, 2023b).

### 1.2. Literature Review

**Parental aggravation and child internalizing problems.** According to parents, feeling aggravated stems mostly from the stress parenthood brings (Bronte-Tinkew et al., 2009). This is especially the case in low-income families. According to Moore and Ehrle, parents are more likely to report feeling aggravated when living in a low-income household (14%) and/or when being a single parent (16%) (Moore & Ehrle, 1999). These statistics show that not only does being a parent alone contribute to parental aggravation, but external stressors, such as financial strain and partner dissolution, also have an impact on parental aggravation (Murphey et al., 2014). When a parent expresses aggravation towards their children, the children may feel responsible and blame themselves for their parents’ negative emotions and, in turn, attempt to mitigate their parents’ distress (The Bowen Center for the Study of the Family [BCSF], 2023a). These acts make children feel more heightened and sensitive to their parents’ dysregulation and increase their vulnerability to develop internalizing problems (The Bowen Center for the Study of the Family [BCSF], 2023a, 2023b). Related research has shown that parental negativity (e.g., hostility) has been linked to putting children at higher risk for internalizing issues (Katsantonis & Symonds, 2023). Suh and Luthar (2020) studied a sample from the 2016 National Survey of Children’s Health, which involved a sample of approximately 36,000 caregivers in the U.S. Their study partly aimed at examining what predicts childhood internalizing problems, with emphasis on parental aggravation and adverse childhood experiences (ACEs) (i.e., parental separation and low socioeconomic status). Both researchers found that internalizing issues were more prominent when parents reported being aggravated (Suh & Luthar, 2020). In addition, parental aggravation had larger effects on child maladjustment than ACEs (Suh & Luthar, 2020), emphasizing how important parenting attitudes and behaviors are for child wellbeing.

**Reciprocal influences between parents and children**. When examining childhood internalizing problems longitudinally using both family systems and socialization theories, we can presume that parent–child dyads have reciprocal influences (Maccoby, 2003). Regarding parent effects, parents who act and/or respond to stressors around their children with a positive, calm demeanor are likely to see similar responses in their children when faced with a stressor (Lim & Shim, 2021) and thus, experience positive family functioning. In comparison, parents who act and/or respond to stressors around their children with a negative, reactive demeanor (perhaps, through verbally aggressing) are likely to see similar responses in their children (Lim & Shim, 2021). Then, when children respond negatively to future stressors (i.e., through internalizing problems), parental aggravation continues, continuing the maladaptive cycle. Regarding child effects, children dealing with internalizing problems could lead to an increase in stress and dysfunction for all family members, but mostly the parents (Suh & Luthar, 2020). This increase in stress and dysfunction creates an influx of parental aggravation behaviors towards children. In response to these behaviors, the children could exhibit more internalizing problems, which dysregulate the family system and further aggravate the parents, thus creating an everlasting cycle of poor wellbeing for children and their families. Taken together, it is possible that not only do parental aggravation influence internalizing problems in children, but their problems can also influence parental aggravation.

**Verbal aggression as a mediating factor**. Parents may display their aggravation through projecting anger towards their children and/or through invalidating their negative emotions (Gawlik & Melnyk, 2022; Nelson et al., 2009). This suggests that aggravated parents may have an increased proclivity to act verbally aggressive towards their children, which can involve yelling, threatening, insulting, and/or criticizing (Gawlik & Melnyk, 2022). Yang et al. found there is a high correlation between parental aggravation and child abuse, which can include verbal aggression (Yang et al., 2022). Since parental verbal aggression is associated with increased internalizing problems in children (Polcari et al., 2014), it is possible that parental aggravation leads to verbal aggression towards their children, which then results in children experiencing heightened internalizing problems. Since parents of children with internalizing disorders experience higher stress (Minkin & Horowitz, 2023), those stressed parents may act verbally aggressive towards their children as a form of coping with aggravation, which unintentionally creates an ongoing cycle of being aggravated towards their children, verbally aggressing, and then their children experiencing heightened anxious/depressive problems. It is also crucial to acknowledge the bidirectional effects; since it can be stressful for parents to watch their children suffer with anxiety and/or depression problems, those parents may start verbally aggressing towards their child(ren) (i.e., insulting, invalidating, etc.) due to feeling aggravated.

**Child gender as a covariate**. The previous literature has suggested that girls have higher prevalences of developing internalizing problems compared to boys (Green et al., 2004). Further, child gender may influence responses to maternal aggravation. Karababa (2024) studied the association between parental hostility and adolescent emotional problems through surveying approximately 1500 adolescents and their cohabiting parents. Karababa found that, when exposed to maternal hostility, adolescent girls reported more emotional problems than boys did. It is possible that children may be influenced more by the parent who shares their same gender. Referring back to FST, children often learn how to cope with stressful situations through observing their parents; therefore, children may be more inclined to adopt the same response to stress as their same-sex parent, whether maladaptive or not. Based on the literature pertaining to parent–child interactions and gender, it would make sense that girls who are exposed to maternal aggravation and verbal aggression would have higher levels of internalizing problems.

### 1.3. The Present Study

Guided by the theoretical perspectives of family systems theory (Bowen, 1966) and socialization theory (Kalmuss & Seltzer, 1989), we addressed the limitations in the previous literature by examining the longitudinal and reciprocal processes of maternal aggravation to child internalizing problems through maternal verbal aggression. Data from the Future of Families & Child Wellbeing Study (FFCWS) were used, as the FFCWS provides several unique advantages. A major limitation in many current studies on this topic is that they used small convenience sampling with a cross-sectional design (Zitzmann et al., 2023). The FFCWS is a longitudinal prospective study, which recruited almost 5000 mothers and children from major cities across the U.S. Further, the FFCWS oversampled low-income single mothers and their children, which allows for the opportunity to focus on this vulnerable population to obtain a better understanding of mental health outcomes among children growing up in these conditions and the challenges they face (Moore & Ehrle, 1999). This could inform intervention programs to better assist these low-income, single mothers and their children to mitigate potential poor parenting practices and child mental health outcomes.

Based on the theories and the literature, using the FFCWS, we hypothesized that there would be bidirectional influences of maternal aggravation and child internalizing problems from childhood to adolescence (H1). We further hypothesized that maternal verbal aggression would mediate the reciprocal associations (H2). Based on the literature, child gender would be considered as a covariate in examining the processes.

## 2. Materials and Methods

### 2.1. Participants and Procedures

The present study used data from the Future of Families & Child Wellbeing Study (FFCWS). For recruitment, FFCWS researchers recruited 4898 children born between the years 1998–2000 from 20 large U.S. cities (population > 200,000) to participate in this longitudinal study. Recruitment methods involved a three-stage stratified sampling process to obtain a nationally representative sample of non-marital U.S. births. Through this sampling method, there was a large inclusion of Black, Hispanic, and low-income families. FFCWS researchers first sampled large cities, then hospitals within those cities, and lastly, births within those hospitals to recruit longitudinal participants (Reichman et al., 2001). Births to unmarried mothers were purposely oversampled, with the ratio being 3:1.

At baseline (Wave I, age 0), data were collected from 3711 nonmarital and 1187 marital births; in total, those births accounted for 4898 infants (*N* = 4898). Mothers were interviewed shortly after birth in the hospital and asked about their marital status; mothers who were “over quota” for a certain marital status (i.e., wed mothers) did not proceed with interviewing. Regarding the sample demographics, most self-reported as Black (47.5%), with a smaller percentage self-reporting as White (21.0%). For ethnicity, most mothers self-reported as not Hispanic or Latino (72.5%), with a smaller percentage self-reporting as Hispanic or Latino (27.3%). Ages of mothers ranged from <18 (9.2%) to >40 (2.3%). The most common ages seen among mothers were ages 19–29 (67.3%). Table 1 provides information regarding all demographics from Wave I.

Since Wave I, six more waves of data were subsequently collected following the children and their parent(s). Each subsequent wave occurred when the child was age 1 (Wave II), 3 (Wave III), 5 (Wave IV), 9 (Wave V), 15 (Wave VI), and 22 (Wave VII). For the present study, because of our focus on the developmental period from childhood to adolescence, Wave III (age 3, *N* = 4231), Wave IV (age 5, *N* = 4139), Wave V (age 9, *N* = 3515), and Wave VI (age 15, *N* = 3580) were assessed to capture child–adolescent developmental trajectories. As a common issue in longitudinal studies, attrition is an issue in the FFCWS. To reduce the potential effect of attrition bias, Full Information Maximum Likelihood (FIML) was used to analyze the data, as it has been shown to produce less biased estimates as compared to other methods, such as listwise deletion, even when data were not missing at random and had a high percentage of missingness (Acock, 2005). The advantage of the large sample provides sufficient power to perform hypothesis testing (MacCallum et al., 1996).

### 2.2. Measures

**Maternal aggravation.** Maternal aggravation was assessed with four items from the Child Development Supplement of the Panel Study of Income Dynamics (PSID-CDS) (Kalmuss & Seltzer, 1989). The PSID-CDS was first developed to examine parental stress caused by changes in employment, income, and/or other factors. Some items in the PSID-CDS were taken from the Parent Stress Inventory (PSI) (Abidin, 1995). The PSI was developed to detect parenting characteristics that fail to promote normal development and functioning in children. The PSID-CDS was created for the National Evaluation of Welfare-to-Work Strategies (NEWWS) to assess the effectiveness of welfare-to-work programs in the United States, including their program impact and outcomes. This measure tapped into negative parental attitudes (i.e., “being a parent is harder than I thought it would be;” “I feel trapped by my responsibilities as a parent;” “I find that taking care of my child is much more work than pleasure” and “I often feel tired, worn out, or exhausted from raising a family”). Each item was answered with a four-point Likert Scale ranging from 1 = *strongly agree* to 4 = *strongly disagree*. Items were reverse-coded and averaged with a higher score representing a higher level of maternal aggravation. The reliability of the scale in the present study was excellent (*α* = 0.99 for Wave III, 0.99 for Wave IV, 0.99 for Wave V, and 0.99 for Wave VI). This measure has been used in previous studies to assess parental aggravation (Annunziato et al., 2007; Yu & Singh, 2012) and has demonstrated sufficient validity and reliability.

**Maternal verbal aggression.** Maternal verbal aggression was assessed through the verbal aggression items from the Parent–Child Conflict Tactics Scales (CTSPC) (Straus et al., 1998). There were five items from Waves III through V. Wave VI had one item that combined two items from Waves III-V into one question, asking about parental behaviors within the past year (e.g., “shouted, yelled, screamed, swore, or cursed”). Each item provided six possible responses, with Waves III through V ranging from 1 = *once* to 6 = *more than 20 times*. The one combined item from Wave VI provided three possible responses, ranging from 1 = *never* to 3 = *often*. A mean score was computed from the items to assess the overall maternal verbal aggression, with a higher score indicating higher maternal verbal aggression. The reliability of the scale used in the present study was excellent (*α* = 0.99 for Wave III, 0.99 for Wave IV, and 0.98 for Wave V). This measure has been used in previous studies to assess psychological (verbal) aggression in parents (Kaiser & Miller-Perrin, 2009; Thakur & Cohen, 2019) and has demonstrated sufficient validity and reliability.

**Child/Adolescent internalizing problems.** Child internalizing problems were assessed through the Child Behavior Checklist (CBCL) (Achenbach, 1992). In this study, we used the anxious/depressed subscale from the CBCL, which was composed of 8 items for Wave III, 14 items for Wave IV, 13 items for Wave V, and 6 items for Wave IV. Items which were consistent across all four waves were “[My] child is nervous and high-strung, [or tense]”, and “[my child] is too fearful [or anxious].” Other items included “[my] child cries a lot” and “[my] child feels worthless or inferior.” Each item provided three possible responses, with Waves III and IV ranging from 0 = *not true* to 2 = *often true*. For Waves V and VI, the coding scheme ranged from 1 = *not true* to 3 = *often true*. A mean score was computed from the items to indicate an overall child internalizing problems score, with a higher score indicated higher internalizing problems. The reliability of the scale used in the present study was acceptable (*α* = 0.64 for Wave III, 0.68 for Wave IV, 0.78 for Wave V, and 0.75 for Wave VI). This measure has been widely used to assess internalizing problems in children and adolescents (Guberman & Manassis, 2011; Thakur & Cohen, 2019) and has demonstrated sufficient validity and reliability.

### 2.3. Analytical Strategies

To test the longitudinal and reciprocal effects of maternal aggravation and anxiety problems from childhood to adolescence and the mediating effects of verbal aggression, a cross-lagged autoregressive model was conducted using AMOS 29. Even though the coding schemes varied a bit across waves for maternal verbal aggression and internalizing problems in the FFCWS, this does not affect autoregressive modeling because the contents of the items were the same, and the purpose of the autoregressive model was to compare the strengths of the paths/associations rather than the mean levels across waves. The model fit was evaluated with the following indices: chi-square (χ^2^), Root Mean Square Error of approximation (RMSEA < 0.05), and with Comparative Fit Index (CFI > 0.95) (Bugental & Grusec, 2007). Child gender (1 = *male*, 2 = *female*) was controlled for.

## 3. Results

### 3.1. Preliminary Analyses

Table 2 presents the correlations among maternal aggravation, verbal aggression, and child internalizing problems across Waves III through VI. The correlations suggested that maternal aggravation, verbal aggression, and child internalizing problems were correlated within waves. For example, the correlation between maternal aggravation and verbal aggression in Wave III was 0.19 (*p* < 0.01), and the correlation between verbal aggression and child internalizing problems in Wave III was 0.17 (*p* < 0.01). There were also significant correlations across waves; for example, the correlation between maternal aggravation in Wave III and verbal aggression in Wave IV was 0.19 (*p* < 0.01), and the correlation between maternal verbal aggression in Wave IV and child internalizing problems in Wave V was 0.10 (*p* < 0.01). The correlation table also shows significance in reciprocal relations between child internalizing problems and maternal verbal aggression and aggravation across waves. For example, the correlation between child internalizing problems in Wave III and maternal verbal aggression in Wave IV was 0.11 (*p* < 0.01). Child internalizing problems in Wave III were also correlated with maternal aggravation in Wave VI (0.14, *p* < 0.01). These correlations showed preliminary support for both our hypotheses.

### 3.2. Hypotheses Testing

**Figure 1:**
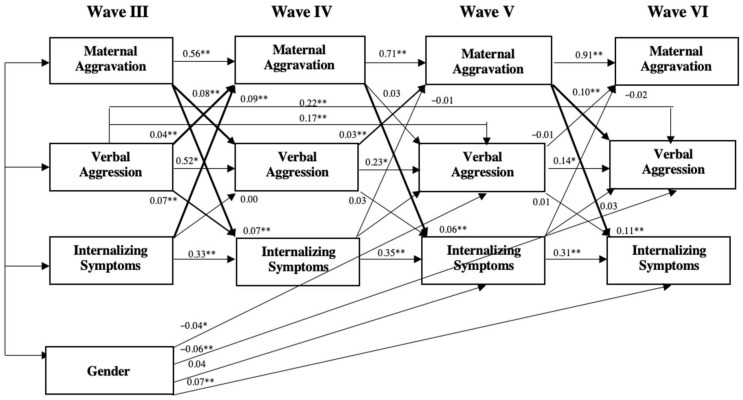
Cross-lagged autoregressive model for maternal aggravation and internalizing problems from childhood to adolescence. *N* = 4898, *χ*^2^ (25) = 72.96, *p* = 0.000; *CFI* = 0.99; *RMSEA* = 0.02, *p-close* = 1.00. Standardized path coefficients are reported. All stability paths were significant (*p* < 0.01). Cross-lagged paths in bold were significant. All paths from gender shown were significant. ** *p* < 0.01, * *p* < 0.05.

Figure 1 shows the cross-lagged autoregressive model of maternal aggravation, verbal aggression, and internalizing problems across the four waves. Residuals were correlated among the variables within each timepoint from Wave IV through Wave VI. Child gender was added as a control variable, and only significant paths from it were included in the final model. For non-adjacent stability paths across waves, only significant ones were included. Standardized path coefficients were reported. Overall, this model showed a relatively good fit: *χ*^2^ (25) = 72.96, *p* = 0.000; *CFI* = 0.99; *RMSEA* = 0.02, and *p-close* = 1.00. All stability paths were significant (e.g., β = 0.56 from maternal aggravation in Wave III to Wave IV). There were two significant paths between non-adjacent constructs—from verbal aggression Waves III to V (β = 0.17, *p* < 0.01) and to VI (β = 0.22, *p* < 0.01). We now turn to the cross-lagged paths of hypothesis testing.

**Testing H1—reciprocal effects**. All three cross-lagged paths from maternal aggravation to internalizing problems at the next wave (e.g., maternal aggravation Wave III to child internalizing problems at Wave IV) were all significant (0.07, 0.06, and 0.11, respectively, *p* < 0.01 for all three paths), demonstrating “parent” effects. On the other hand, upon exploring the effects of child internalizing problems on maternal aggravation, only child internalizing problems at Wave III had a significant path to maternal aggravation at Wave IV (*β* = 0.09, *p* < 0.01), suggesting limited “child” effects.

**Testing H2—mediation effects.** Maternal aggravation at Wave III had a significant path to verbal aggression at Wave IV (β = 0.08, *p* < 0.01), and maternal aggravation at Wave V had a significant path to verbal aggression at Wave VI (β = 0.10, *p* < 0.01). However, other than from verbal aggression at Wave III to internalizing problems at Wave IV (β = 0.07, *p* < 0.05), the other two paths from verbal aggression to subsequent internalizing problems were not significant. Regarding the direction from child internalizing problems to maternal aggravation through verbal aggression, only verbal aggression at Wave III to maternal aggravation at Wave IV (β = 0.04, *p* < 0.01) and verbal aggression at Wave IV to maternal aggravation at Wave V (β = 0.03, *p* = 0.05) were significant; no path from child internalizing problems to subsequent verbal aggression was significant. As a result, mediation was not supported in either direction.

Gender (1 = male, 2 = female) was tested in the cross-lagged autoregressive model as a covariate, and significant paths were included in the final model. Four significant paths were found and included. Specifically, gender was significantly related to verbal aggression at Wave IV (β = −0.04, *p* < 0.05) and Wave VI (β = −0.06, *p* < 0.01), suggesting lower levels of maternal aggravation and verbal aggression toward girls. The path from gender to internalizing problems at Wave V (β = −0.04, *p* < 0.05) and Wave VI (β = 0.07, *p* < 0.01) presented mixed results.

## 4. Discussion

Within recent years, there has been a drastic rise in internalizing problems in children; so much so that this increase has been deemed a public health concern. Consequential future outcomes can occur for children who reach the diagnostic criteria for an internalizing disorder, including poor work performance, academic achievement, and social relationships (National Academies of Sciences, Engineering, and Medicine, 2016; World Health Organization [WHO], 2024). With anxious and depressive symptoms being the most common internalizing issues among children (Centers for Disease Control and Prevention [CDC], 2023; Chiu et al., 2016), family researchers and practitioners have been questioning what causes these increases in childhood internalizing problems. As parents have major influences on child psychoemotional wellbeing (Gawlik & Melnyk, 2022), researchers looked into maladaptive parenting behaviors for more insight.

This current study expands the current literature on the effects of negative parenting attitudes (i.e., aggravation) and behaviors (i.e., verbal aggression) on child wellbeing regarding the development of internalizing problems, as well as possible reciprocal effects. Based on the theoretical frameworks of family systems theory and family socialization theory, along with the related literature, we proposed longitudinal and bidirectional influences of maternal aggravation and child internalizing problems (H1) and that maternal verbal aggression would mediate those associations (H2). With a sample of 4898 mothers and children from low-income families from the national dataset of the FFCWS, results from our cross-lagged autoregressive model provided some support to the hypotheses, but also mixed findings.

### 4.1. Maternal Aggravation on Child Internalizing Problems

Theories and the previous literature have linked negative parenting attitudes with the development of internalizing problems in children. Based on the works of Moore and Ehrle (1999), parents were more likely to report feeling aggravated when living in a low-income household (14%) and/or when being a single parent (16%), which was consistent with our current sample. With this knowledge, parents may show aggravation towards their children as a maladaptive way of coping with their external stressors. Researchers have also stated that internalizing problems were more prominent among children whose parents acted aggravated and/or hostile towards them (Katsantonis & Symonds, 2023; Suh & Luthar, 2020). These findings were consistent with our hypothesis that aggravation from mothers is linked to childhood internalizing problems (i.e., “parent effects”).

Our cross-lagged autoregressive model found significant, direct paths of maternal aggravation to child internalizing problems across all waves, supporting the parent effects portion of our H1 while highlighting the important role of maternal aggravation. Our findings showed direct influences of maternal aggravation on child internalizing problems, which are consistent with the theoretical framework of FST (Bowen, 1966). For instance, displays of parental aggravation can disrupt the family unit, making children feel more heightened and sensitive to their parents’ dysregulation; thus, increasing their vulnerability to develop internalizing problems (The Bowen Center for the Study of the Family [BCSF], 2023a, 2023b). “Parent effects” are also consistent with family socialization theory (Kalmuss & Seltzer, 1989). For instance, children who observe aggravated behaviors from their parents may model those same poor emotional regulation skills. This is crucial, as poor emotional regulation skills are linked to childhood internalizing problems (Kalmuss & Seltzer, 1989; Loevaas et al., 2018). Overall, both theories supported the direct effects from H1 in this current study. Based on both theories and the previous literature, it is suggested that maternal aggression influences internalizing problems from early childhood to adolescence, emphasizing the importance of researching the long-term effects of maladaptive mothering processes on child anxiety and depression. This suggests that children may have lower risks of developing internalizing problems when their mothers display positive attitudes towards them, versus negative attitudes.

### 4.2. Child Internalizing Problems on Maternal Aggravation

In addition to maternal aggravation leading to child internalizing problems, the current literature provided limited evidence that there could be possible reciprocal effects (i.e., “child effects”) of internalizing problems on maternal aggravation (Maccoby, 2003), at least during the early stages of child development. Minkin and Horowitz (2023) found that parents of children with internalizing problems experience higher stress. In addition, Woodman et al. (2015) found a correlation between child internalizing problems and high stress in parents, which can turn into aggravation.

In our study, we found limited evidence of “child effects”, in that child internalizing problems were found to be directly related to maternal aggravation only during earlier stages of development (i.e., Wave III, when the child was three years old). The implications could be that children have greater influences on maternal wellbeing when younger compared to when they are older. Since younger children have higher support needs and sometimes experience unstable emotions, in response to such demands, mothers may experience heightened stress.

### 4.3. Verbal Aggression as a Mediator

Research has suggested that aggravated parents may have an increased proclivity to act verbally aggressive towards their children (Gawlik & Melnyk, 2022; Nelson et al., 2009). In this case, verbal aggression is viewed as a negative, outward response to aggravation. Verbal aggression, which includes yelling, threatening, insulting, and/or criticizing someone (Gawlik & Melnyk, 2022), has been found to be associated with increased problems of anxiety and depression in children (Polcari et al., 2014). Based on the ongoing literature, the present study proposed that verbal aggression mediated the linkage between maternal aggravation influencing child internalizing problems, and vice versa. 

The findings from this study, however, did not support this hypothesis. Even though our model revealed significant paths from maternal aggravation at Waves III and V to subsequent verbal aggression and verbal aggression at Wave III to internalizing problems at Wave IV, mediation for “parent” effects was not established. Similarly, even though verbal aggression at Wave III and Wave IV was significantly related to maternal aggravation in subsequent waves, no path from child internalizing problems to verbal aggression was significant; therefore, mediation for “child” effects was not established. While it suggested a direct influence of maternal aggravation rather than from verbal aggression, this finding was not consistent with some previous studies (Gawlik & Melnyk, 2022; Polcari et al., 2014). We believe this null finding could be due to methodological limitations in this study, such as the specific sample and the measurements, rather than disproving the theories. These are further discussed in Section 4.6.

### 4.4. Gender as a Covariate

There were some interesting findings about gender differences. Girls were associated with lower levels of maternal verbal aggression. This could be due to a plethora of reasons; perhaps, because girls were viewed to be more emotional compared to boys (Brody & Hall, 2008) and received less verbal aggression from parents to spare their feelings. Alongside this finding, girls were more positively associated with internalizing problems at Wave VI. This was not a surprising find, as the literature did state that girls do have a higher prevalence of developing internalizing problems compared to boys (Green et al., 2004). The negative association between gender and internalizing problems at Wave V, however, was inconsistent with the literature.

### 4.5. Practical Contributions

Our findings were important and supplemental to the ongoing literature about the effects of parental behaviors on child wellbeing. This study bridged a gap in the literature in examining the effects of maternal aggravation on child internalizing problems. As childhood internalizing issues are becoming more prevalent, this study could help navigate family practitioners of all domains (i.e., physicians, psychologists, etc.) in understanding that sometimes, the onset of child internalizing problems starts within the home. With this understanding, more practitioners could consider adopting a stronger family systems lens when providing treatments and/or interventions. Overall, with caution of measurement issues in mind, our findings seem to suggest stronger evidence of “parent” effects than “child” effects and direct effects than mediating effects.

### 4.6. Limitations and Future Directions

Despite the strengths of this longitudinal study with a national dataset, there were a few limitations in this study that must be addressed. First, regarding the sample, although the intentional focus on children from low-income, single mothers in the FFCWS provided unique insights into the functions and processes of these families, the sample was not representative of the broader population, and therefore, the findings should not be overgeneralized. Further, we used data from the FFCWS when children were at ages 3, 5, 9, and 15. Data during certain critical transition periods in between those ages were not available in the FFCWS, and therefore, we were not able to analyze the variables of interests at those periods. In addition, attrition was an issue. Even though FIML was used in modeling, the generalizability was limited, and the findings should be interpreted with caution.

Second, regarding the measurements, the number of items was inconsistent across the four waves for verbal aggression and internalizing problems. For verbal aggression, two items in earlier waves were combined into one at Wave VI. For CBCL, the unequal representation of items was partially due to the FFCWS needing items to maintain age-appropriateness across waves (e.g., “[child] clings to adults” in Wave III would not be as applicable in Wave IV). Although FFCWS researchers tried to maintain content validity across waves, the difference in the number of items posed a potential issue for testing constructs across time. Though the difference in the number of items did not prevent us from using a cross-lagged model to investigate the strength of correlations across time, these changes in measurements nevertheless could have affected the comparison of the estimation of the stability and cross-lagged associations across waves. The inconsistency in measures of verbal aggression and child internalizing problems across waves may also have contributed to the null findings of mediation from child internalizing problems through verbal aggression. Despite the limitations in these measures in the FFCWS, we did our best to adapt these measures while holding their content validity. Future studies should employ measures that have a sufficient number of items that are consistent across time, which allow for a more robust estimation of the stability and change in these constructs over time.

Third, it should be noted that all measures were reported by mothers, which could have produced reporter bias. When mothers self-reported their aggravation and verbal aggression, they may have felt inclined to report minimal levels of aggravation and aggression towards their children due to social desirability (Bornstein et al., 2014). When mothers reported their child’s problems, there was a chance of reporting error or bias (Najman et al., 2001), especially in relation to internalizing problems, where problems could be masked and cannot be easily observed. Despite mothers being around their children often, it could have been difficult to know what their children were internally going through. Further, when testing associations among variables all reported by a single informant (i.e., mothers), these associations could be inflated. Future studies should consider using multi-informant methods and possibly including observational data.

Fourth, this study only considered the potential mediating effect of maternal verbal aggression. The null finding on mediation may also suggest that there could be other possible mediating mechanisms. Researchers should look into other possible mediators between maternal aggravation and child internalizing problems. For instance, mothers may display their feelings of aggravation in other ways than verbally aggressing. Thus, other maladaptive maternal behaviors, such as physical aggression, may serve as a better mediator.

Fifth, due to the complexity of the cross-lagged autoregressive model, this study only considered gender as a covariate. Future studies should explore the role of other possible theory-driven covariates, such as mothers’ relationship partner status and living arrangement (e.g., whether the mother is cohabiting with a partner could influence child outcomes through having another supportive parental figure in the household). The potential moderating roles of these factors should also be considered. In addition, future studies could further take advantage of the rich FFCWS dataset. For example, future studies could expand the dependent variables to include both child internalizing problems and externalizing problems. This would allow researchers to obtain a thorough understanding of which childhood psychoemotional difficulties are associated with maternal maltreatment. Lastly, the FFCWS recently published results from Wave VII (age 22). Researchers who wish to continue studying childhood outcomes past adolescence and into young adulthood are encouraged to view that dataset.

## 5. Conclusions

In summary, this study utilized a sample of low-income, single mothers and their children to examine the bidirectional influences of maternal aggravation and child internalizing problems mediated by verbal aggression. Using family systems theory, family socialization theory, and the related literature, the results showed that there were direct paths from maternal aggravation to child internalizing problems. Reciprocal effects of childhood internalizing problems influencing maternal aggression were found only in younger children. No mediation by maternal verbal aggression was found. Furthermore, the current study found significant gender differences; girls were less likely to endure verbal aggression but more likely to develop internalizing problems in adolescence. As childhood internalizing problems are on the rise, these findings could further assist family practitioners in understanding the direct causes of these problems and thus, provide the best treatments and/or interventions for children and their families. Specifically, given the saliency of maternal aggravation, policies and programs should aim to improve the lives of low-income single mothers (e.g., living conditions, safe environment, financial assistance, and social support), which alleviates stress to reduce negative parenting attitudes and behaviors. Parenting programs can also involve the whole family system and directly tackle negative parenting behaviors with the goal of improving child wellbeing.

## Figures and Tables

**Table 1:** Demographic information.

Variable	N	Percent
Child Sex		
Boy	2556	52.2%
Girl	2341	47.8%
Mother Age		
Below 18	450	9.2%
19–29	3292	67.3%
30–39	1038	21.2%
40–49	114	2.3%
Mother Race		
White	1030	21.0%
Black	2326	47.5%
Other	1530	31.3%
Mother Ethnicity		
Hispanic or Latino	1336	27.3%
Not Hispanic or Latino	3550	72.5%
Mother Educational level		
Less than high school	1699	34.7%
High school or equivalent	1480	30.2%
Some college	1189	24.3%
College or graduate school	524	10.7%

*Note. N* = 4898.

**Table 2:** Correlations for study variables.

Variables	1	2	3	4	5	6	7	8	9	10	11	12
1. Maternal Aggravation III												
2. M. Aggravation IV	0.59 **											
3. M. Aggravation V	0.42 **	0.49 **										
4. M. Aggravation VI	0.39 **	0.46 **	0.49 **									
5. Verbal Aggression III	0.19 **	0.16 **	0.15 **	0.12 **								
6. V. Aggression IV	0.19 **	0.19 **	0.18 **	0.13 **	0.53 **							
7. V. Aggression V	0.07 **	0.09 **	0.11 **	0.09 **	0.27 **	0.29 **						
8. V. Aggression VI	0.10 **	0.12 **	0.16 **	0.24 **	0.28 **	0.34 **	0.24 **					
9. Internalizing III	0.17 **	0.18 **	0.15 **	0.14 **	0.17 **	0.11 **	−0.03	0.07 **				
10. Internalizing IV	0.14 **	0.22 **	0.17 **	0.12 **	0.14 **	0.19 **	0.03	0.07 **	0.35 **			
11. Internalizing V	0.09 *	0.14 **	0.19 **	0.15 **	0.10 **	0.10 **	0.11 **	0.09 **	0.20 **	0.36 **		
12. Internalizing VI	0.12 **	0.15 **	0.17 **	0.24 **	0.05 **	0.06 **	0.05 *	0.15 **	0.14 **	0.23 **	0.35 **	
Mean (M)	2.26	2.18	2.03	2.06	1.60	1.74	3.06	1.83	0.45	0.24	1.20	1.25
Standard Deviation (SD)	0.67	0.69	0.68	0.70	0.98	1.02	1.36	0.65	0.32	0.23	0.22	0.31

*Note*. *N* = 4898. * *p* < 0.05; ** *p* < 0.01. Two-tailed tests.

## Data Availability

The Future of Families & Child Wellbeing Study (FFCWS) data are publicly available at https://ffcws.princeton.edu/ (assessed on 16 January 2026).
